# Gastric Outlet Obstruction Caused by External Compression by a Giant Gallstone Treated With Laparoscopic Cholecystectomy: A Case Report

**DOI:** 10.7759/cureus.104157

**Published:** 2026-02-23

**Authors:** Ayşegül Bahar Özocak

**Affiliations:** 1 General Surgery, Edirne Keşan State Hospital, Edirne, TUR

**Keywords:** bouveret syndrome, external compression, gastric outlet obstruction, giant gallstone, laparoscopic cholecystectomy

## Abstract

Gastric outlet obstruction (GOO) caused by gallstones is classically described as Bouveret’s syndrome and results from the impaction of a migrated stone through a bilioenteric fistula. GOO due to external compression by a non-migrated gallstone is exceptionally rare. We report the case of a 71-year-old male presenting with recurrent projectile vomiting and epigastric distension. Computed tomography revealed marked gastric dilatation caused by extrinsic compression of the duodenum from a giant gallstone measuring approximately 8 cm, without evidence of fistula or inflammation. Initial conservative management provided temporary relief; however, symptoms recurred, and the patient underwent emergency laparoscopic cholecystectomy. Intraoperatively, the gallbladder containing the giant gallstone was found to be externally compressing the duodenum, with intact anatomical planes and no fistula. The postoperative course was uneventful, and oral intake was successfully resumed. This case highlights a rare mechanism of gastric outlet obstruction caused by external compression from a giant gallstone and demonstrates that laparoscopic cholecystectomy can be a safe and effective treatment option, even in the presence of very large gallstones.

## Introduction

Gastric outlet obstruction caused by gallstones is most commonly referred to as Bouveret’s syndrome and typically results from impaction of a migrated giant gallstone within the duodenum [1-3]. In contrast, gastric outlet obstruction due to external compression by a non-migrated gallstone represents an exceptionally rare and scarcely described entity. The laparoscopic management of giant gallstones may be technically challenging because of their size and the presence of chronic inflammatory changes [4]. Nevertheless, several reports have demonstrated that laparoscopic cholecystectomy can be performed successfully even in cases involving large gallstones [5,6]. In this report, we present a case of gastric outlet obstruction caused by external compression from a giant gallstone measuring approximately 8 cm, which was successfully treated with laparoscopic cholecystectomy.

## Case presentation

**Figure 1:**
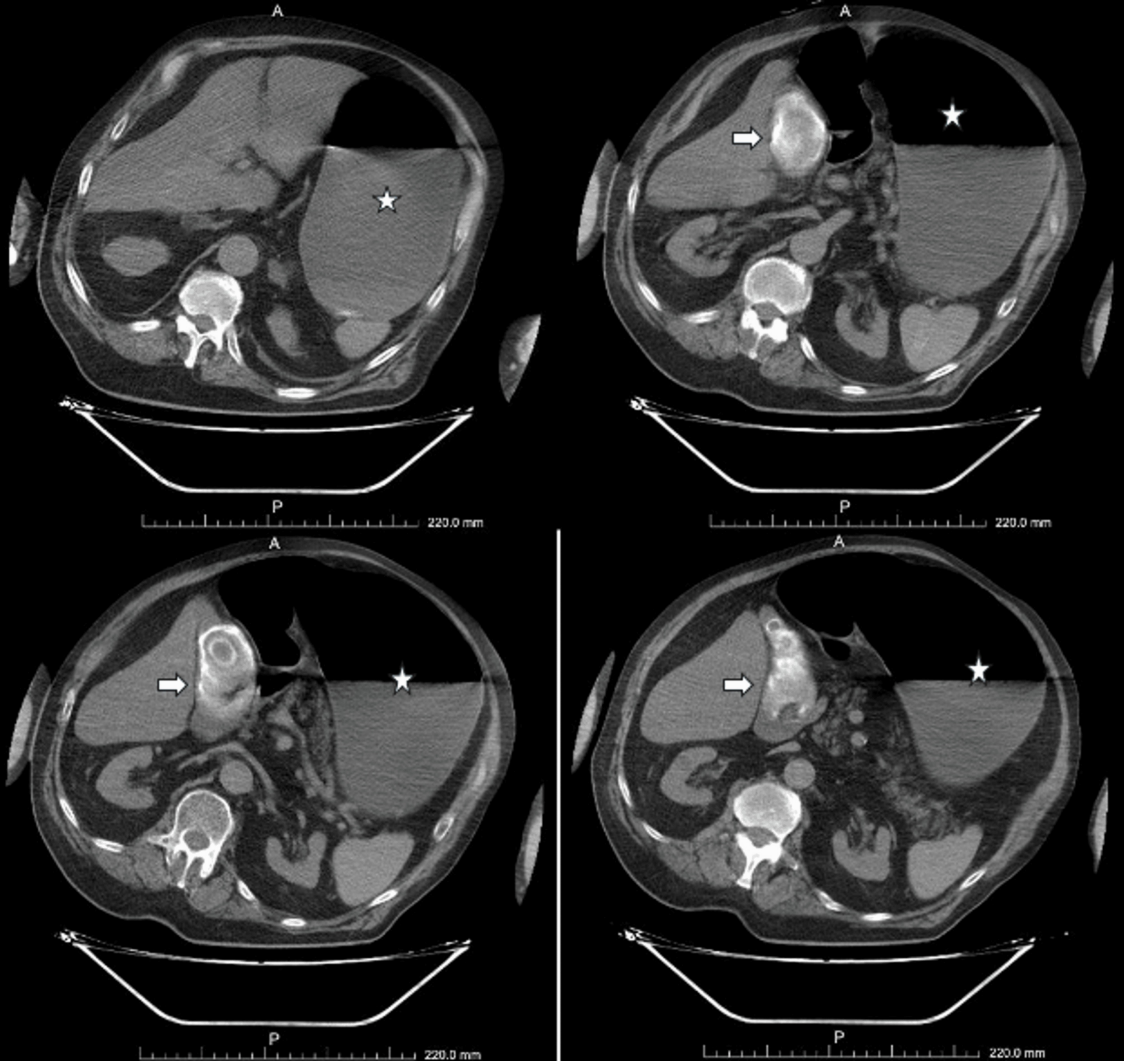
Preoperative abdominal computed tomography demonstrating marked gastric dilatation with significant gaseous and fluid distension. The arrow indicates extrinsic compression of the duodenum by the giant gallstone within the gallbladder. The asterisk (*) marks the markedly distended stomach.

A 71-year-old male patient was admitted to our unit from the coronary intensive care unit for diuresis due to heart failure and was referred to our service because of recurrent projectile vomiting. The patient reported vomiting and was observed to vomit large volumes of gastric contents. Physical examination revealed marked epigastric distension. Abdominal computed tomography (CT) demonstrated significant gaseous and fluid distension of the stomach. (Figure 1) The small intestine and colon were normal, with no evidence of distal intestinal obstruction. Multiple gallstones were identified within the gallbladder, the largest measuring 80 mm in diameter, and the gallbladder width was approximately 50 mm. There were no radiological signs of gallbladder inflammation or a cholecystoduodenal fistula (Figure 1).

A nasogastric (NG) tube was inserted, resulting in the drainage of a large volume of gastric contents and gas. Metoclopramide was initiated for prokinetic support. The patient’s medical history revealed previously diagnosed gallstones; however, he had declined surgical intervention due to the absence of symptoms at that time. Following NG decompression, the patient’s symptoms improved, and NG output gradually decreased. Given the high anesthetic risk associated with his comorbidities, an initial conservative management strategy was adopted. Enteral intake was gradually reintroduced with intermittent NG clamping, and the NG tube was removed once the patient tolerated oral intake.

**Figure 2:**
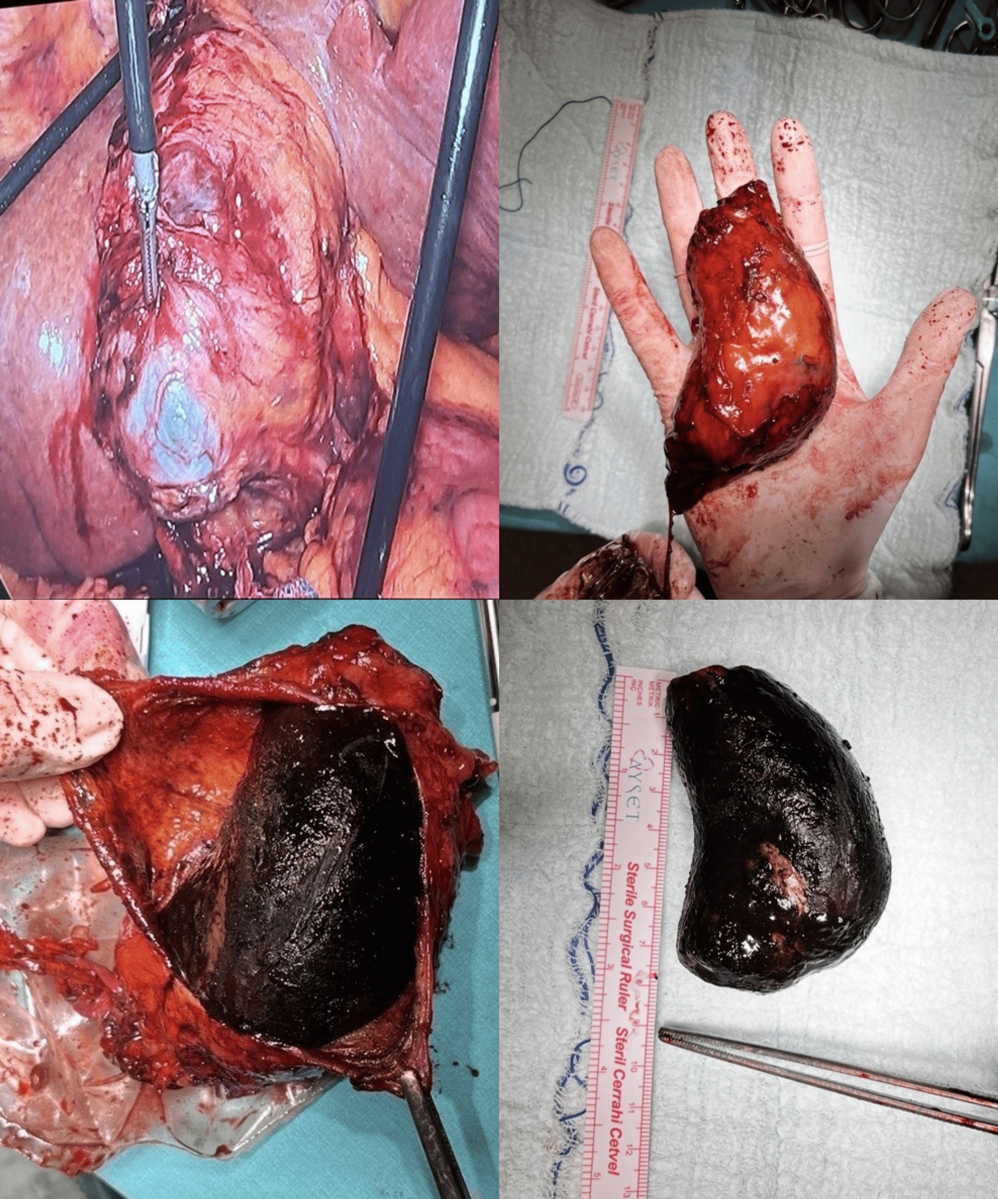
Intraoperative laparoscopic views demonstrating external compression of the duodenum by the gallbladder and careful dissection with preserved anatomical planes, followed by the resected gallbladder specimen containing a giant gallstone measuring approximately 8 cm.

Five days later, the patient developed recurrent vomiting and was re-evaluated. Repeat CT imaging demonstrated persistent gastric outlet obstruction caused by extrinsic compression of the duodenum by the gallstone. Surgical intervention was therefore indicated. The patient underwent emergency laparoscopic cholecystectomy. Intraoperatively, the gallbladder and the giant gallstone were found to be compressing the duodenum externally. Careful dissection confirmed the absence of a fistula, with preserved anatomical planes, consistent with the preoperative imaging findings. Despite technical challenges related to the size of the gallstone, the procedure was completed laparoscopically. Due to the large size of the gallbladder and the giant stone, the specimen was placed in an endoscopic retrieval bag and extracted through a slightly extended umbilical port incision without fragmentation of the stone. Intraoperative findings and the resected specimen are shown in Figure 2.

On postoperative day one, NG output decreased significantly, allowing NG clamping and initiation of clear liquids. On postoperative day two, a soft diet was commenced, and the NG tube was removed. On postoperative day three, the patient progressed to a regular diet, which he tolerated without difficulty.

## Discussion

Gallstone disease is a common condition that may lead to a wide range of complications. Although gallstone size varies considerably, stones approaching 8 cm in diameter, as observed in this case, have been reported only rarely in the literature [7,8]. The distinctive feature of this case is the development of gastric outlet obstruction due to extrinsic duodenal compression by a giant gallstone, in the absence of associated inflammation or fistula formation.

While open surgery has traditionally been recommended for the management of giant gallstones, this case demonstrates that laparoscopic cholecystectomy can be a safe and effective treatment option, even for stones of this size, when performed with careful dissection and meticulous surgical technique. Although gastric outlet obstruction due to gallstones is classically seen in Bouveret syndrome via bilioenteric fistula formation, there are rare case reports in the literature in which external compression by a large, non-migrated gallstone caused obstruction in the absence of a fistula [9]. This mechanism is exceptionally uncommon, making the current case notable due to the size of the stone (80 mm) and the lack of any fistula or gallstone migration.

## Conclusions

Gastric outlet obstruction caused by external compression from a giant, non-migrated gallstone is an exceptionally rare entity. This case highlights the importance of considering gallstone-related extrinsic compression in the differential diagnosis of gastric outlet obstruction. Moreover, it demonstrates that laparoscopic cholecystectomy can be safely and effectively performed in selected patients, even in the presence of very large gallstones.
